# Intraepithelial dendritic cells and sensory nerves are structurally associated and functional interdependent in the cornea

**DOI:** 10.1038/srep36414

**Published:** 2016-11-02

**Authors:** Nan Gao, Patrick Lee, Fu-Shin Yu

**Affiliations:** 1 Departments of Ophthalmology, Anatomy and Cell Biology, Wayne State University School of Medicine, Detroit, Michigan 48201, USA

## Abstract

The corneal epithelium consists of stratified epithelial cells, sparsely interspersed with dendritic cells (DCs) and a dense layer of sensory axons. We sought to assess the structural and functional correlation of DCs and sensory nerves. Two morphologically different DCs, dendriform and round-shaped, were detected in the corneal epithelium. The dendriform DCs were located at the sub-basal space where the nerve plexus resides, with DC dendrites crossing several nerve endings. The round-shaped DCs were closely associated with nerve fiber branching points, penetrating the basement membrane and reaching into the stroma. Phenotypically, the round-shaped DCs were CD86 positive. Trigeminal denervation resulted in epithelial defects with or without total tarsorrhaphy, decreased tear secretion, and the loss of dendriform DCs at the ocular surface. Local DC depletion resulted in a significant decrease in corneal sensitivity, an increase in epithelial defects, and a reduced density of nerve endings at the center of the cornea. Post-wound nerve regeneration was also delayed in the DC-depleted corneas. Taken together, our data show that DCs and sensory nerves are located in close proximity. DCs may play a role in epithelium innervation by accompanying the sensory nerve fibers in crossing the basement membrane and branching into nerve endings.

The cornea is the most heavily innerved tissue in the body primarily supplied by small-diameter C-fiber sensory neurons, with densely distributed sensory nerve endings in the sub-basal space of the epithelium, termed the sub-basal nerve plexus^1^. Most corneal nerve fibers are sensory in origin and are derived from the ophthalmic branch of the trigeminal nerve. The sensory nerves are responsible for sensations of dryness, temperature, touch, and pain, and play important roles in the blink reflex, wound healing, and tear production^2^. Many ocular and systemic diseases can adversely affect corneal sensory nerves and consequently impair their function, with vision loss being the inevitable consequence of severe corneal neurotrophic ulceration^3^,^4^,^5^.

Axons from the trigeminal ganglion terminate in delicate endings among the epithelial cells of the cornea^6^. Nerve bundles enter the cornea from the periphery in a radial fashion. The fibers travel parallel to the corneal surface into the anterior third of the stroma, losing their perineurium and myelin sheaths within approximately 1 mm away from the limbus^6^,^7^. The stromal nerve fibers underneath the basement membrane, one component of Bowman’s layer in humans, turn abruptly 90° and proceed towards the corneal surface. After penetrating the basement membrane, they then abruptly turn 90° once more and continue parallel to the corneal surface, further dividing into smaller fibers and then endings with an extraordinarily high innervation density of the corneal epithelium. To date, the cellular mechanisms underlying epithelium innervation, i.e., how the unmyelinated nerve fibers penetrate through the basement membrane, remain elusive^8^.

Dendritic cells (DCs) are diverse and specialized hematopoietic cells serving as an essential bridge between the innate and adaptive immune systems^9^,^10^. Conventional DCs line the tissues of the body exposed to the exterior environment, such as the skin and the epithelia of the lung^11^, gut^12^, and cornea^13^, where they survey the tissues for invading pathogens or the emergence of pro-inflammatory stimuli^14^. In the cornea, it is increasingly clear that while macrophages have been found only to occupy the posterior stroma, DCs reside in both the stroma and the epithelium, with phenotypically different subtypes^15^,^16^,^17^,^18^. In the corneal epithelium, DCs residing at the basal epithelial layer are more numerous in the peripheral versus central cornea^19^. Some of the DCs at the central cornea insert processes between epithelial cells, similar to that of the vertically-oriented sensory nerve endings. These processes serve to sample antigens from the environment^19^,^20^.

Both sensory nerves and DCs function as sentinels at the mucosal surface of the cornea; while sensory nerves utilize nociceptors to sense chemical and temperature changes, DCs use pattern recognizing receptors such as Toll-like receptors to detect “foreigners”. They are also located in close proximity to one another, within the spaces between the basement membrane and the epithelial sheet in the cornea. While there have been no studies examining the connections of sensory nerve axons and DCs in the cornea, a 2009 study revealed that DCs were in close physical association with sensory nerves and T-cells^21^. The nerve-contacting DCs induced T cell proliferation only in the airways of mice with allergic inflammation but not in the controls^22^. More recently, it was reported that DCs interact with sensory nerves during allergic airway inflammation in a calcitonin gene-related peptide-related manner^23^. Using streptozotocin-induced type I diabetic mouse model, we recently demonstrated that DCs mediate sensory nerve innervation and regeneration through CNTF; diabetes reduces the population of DCs in unwounded and wounded corneas, resulting in a decrease in CNTF concentration and impaired sensory nerve innervation and regeneration.

In the present study, we focused on how these two sentinels, DCs and sensory nerves, influence each other structurally and functionally in tissue homeostasis and in response to wounding. We demonstrated that trigeminal denervation of the cornea resulted in the loss of DCs while local depletion of DCs resulted in altered innervation of the corneas, suggesting that within the epithelial platform they are structurally connected and may have coordinated actions in response to wounding. We also identified an unprecedented role of DCs: accompanying sensory nerve fibers to penetrate the basement membrane and innervate the epithelia.

## Results

### Intraepithelial DCs,sub-basal nerve endings, and epithelium-innervating fibers in the cornea

**Figure 1:**
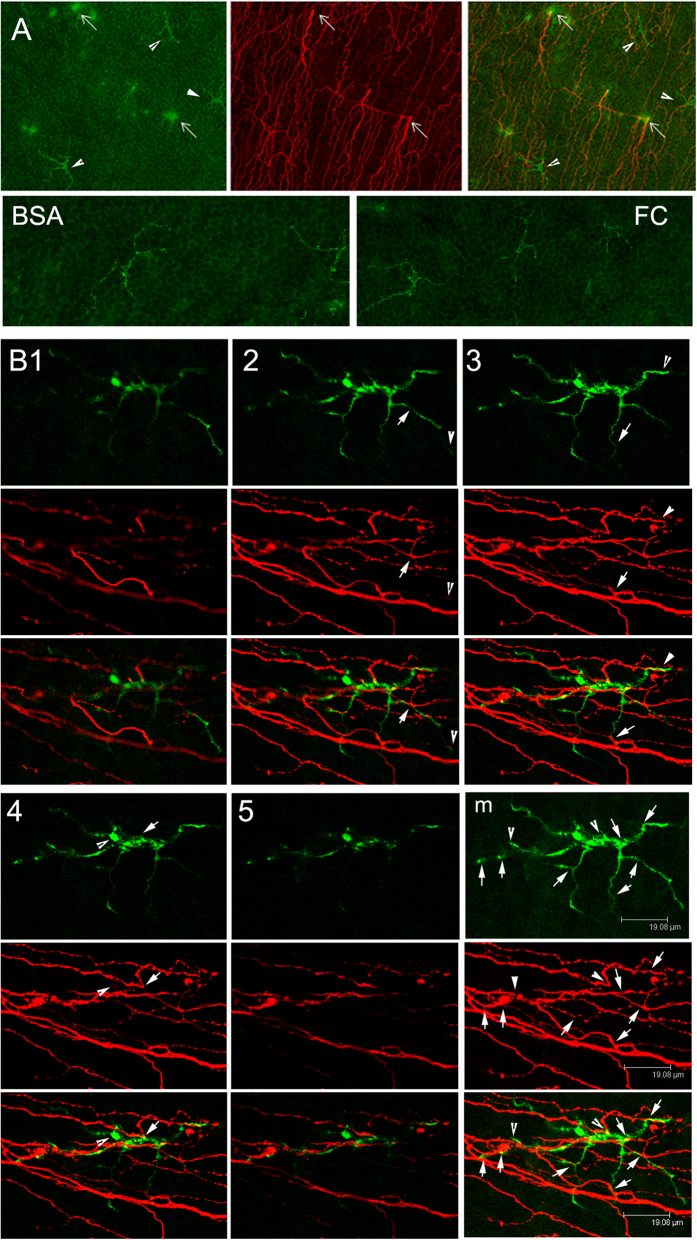
Corneal intraepithelial dendritic cells (DCs) and sensory nerves (I): dendriform DC and sensory nerve endings. Double-staining of a whole-mount specimen (from a naive animal) for nerves (β-tubulin III) and DCs (CD11c) in the cornea, showing typical and atypical DC morphology (dendriform, arrowheads, and round-shaped, arrows) and sensory nerve fibers and endings. Cryostat sections of naive cornea injected mouse corneas were immunostained with antibodies against β-tubulin III and CD11c in the cornea 24 hpw with DAPI in mounting media to illustrate nuclei (blue) (**A**). Optical sections of WMCM from apical to basal direction showing a dendriform DC stained with CD11c (green), sensory nerve endings stained with β-tubulin III (red), and superimposed images in the sub-basal nerve plexus (**B**). Note the DC and Sensory nerve endings appear (B1) and disappear (B5) in the same sections. Arrows and arrowheads in D2–4: corresponding CD11c and β tubulin III co-localization sites. Bm: merged and superimposed images, arrows, potential interconnecting sites; arrowheads, DC cell body co-localizing with linking several nerve endings. The results are representative of 5 independent experiments, 3 corneas each.

We assessed localization of DCs and sensory nerve in mouse corneas using whole mount confocal microscopy (WMCM). CD11c staining revealed two types DCs differing in morphology: dendriform (arrowheads) and round-shaped (arrows) (Fig. 1A). Figure 1B shows 5 optical sections of WMCM, revealing a dendriform DC co-localized with several nerve endings. The merged and superimposed image of CD11c and tubulin III (Fig. 1Bm) showed many co-localization sites (arrows) where sensory endings crossed and/or turned, and a stretch of DC/sensory nerve co-localization (between two arrowheads).

**Figure 2:**
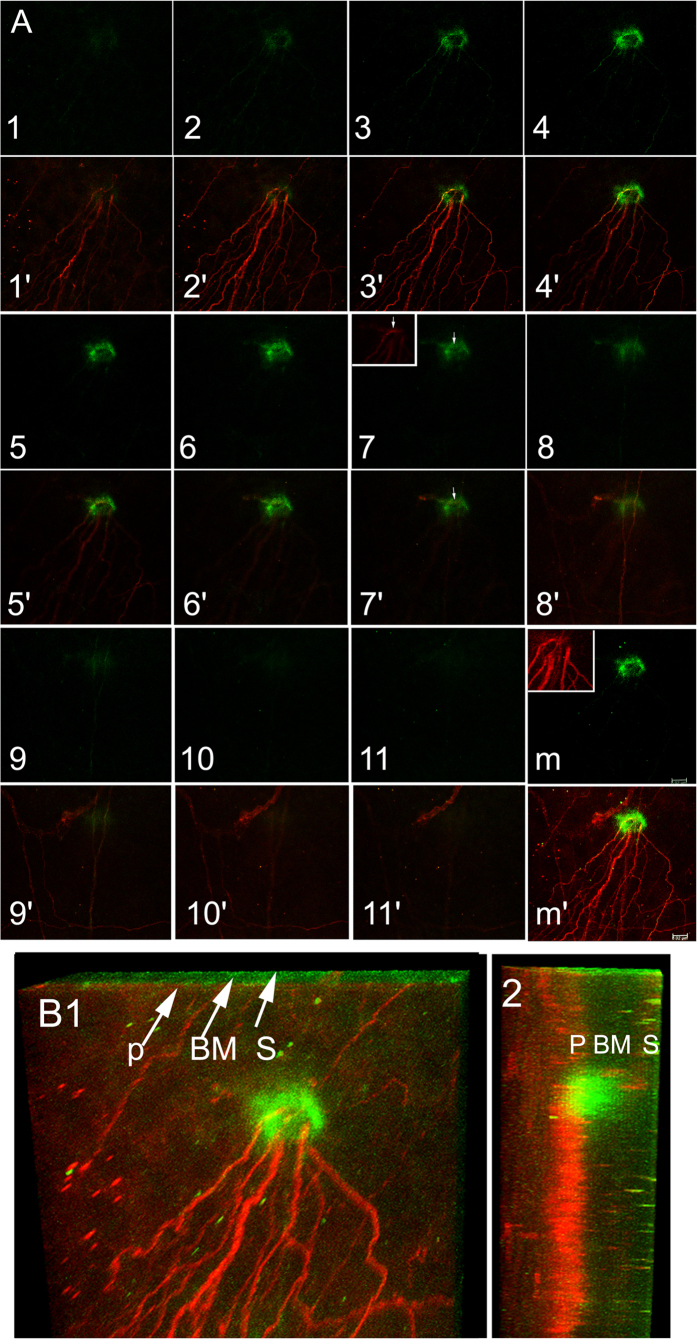
Corneal intraepithelial dendritic cells (DCs) and sensory nerves (II): round-shaped DC and epithelium innervation. Optical sections of a round-shaped DC stained with CD11c (numbers, from apical to basal direction) and superimposed on nerve fiber/ending staining (numbers’) **(A)**. Note a voided space of CD11c staining in the DC (arrows, C7 and 7′) that is similar to the shape of the strong β-tubulin III staining (insert in C7). Panels (m) merged CD11c staining (m) and nerve fiber branching point (inert in m) showing CD11c staining wraps around a branching nerve fiber, with an opening where axons extend through and innervate the epithelium. Optical sections shown in **A** from 2 channel confocal imaging were digitally rendered in 3D imaging for a bird’s eye- (B1) and side-view (B2) **(B)** P, subbasal plexus; BM, basement membrane; S, stroma. Note localization of apical part of the DC within the nerve plexus and middle part penetrating the basement membrane with basal end reached the stroma. The results are representative of 5 independent experiments, 3 corneas each.

Figure 2 shows the co-localization of a round-shaped DC (green) and sensory nerve fibers/endings (red). At the sub-basal plexus layer (sections 1 to 4), several sensory nerve fibers/endings initiated from a CD11c positive cell and radiated towards the center of the cornea. At sections 5–8, while CD11c staining remained strong, nerve branches began to disappear and stromal nerve fibers started to appear. At the stromal layer (sections 9–11), the intensity of CD11c staining decreased but was still detectable with stroma nerve fibers nearby. The merged images of all optical sections showed that a DC wraps around a branching nerve fiber with several axons that extended towards the center of the cornea. Moreover, the side view reveals the DC extends from the sub-basal space into the stroma (B1 and 2).

**Figure 3:**
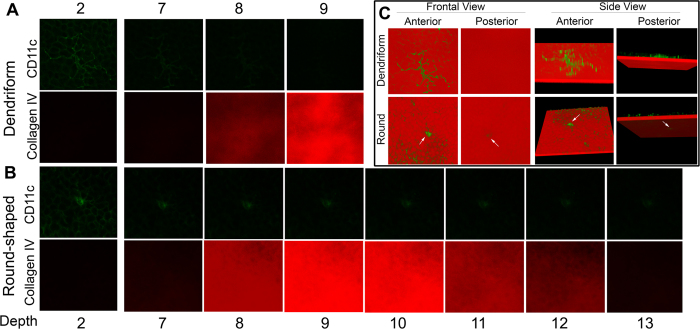
Dendritic cells that accompany sensory nerve fibers to cross the basal membrane in mouse corneas. Double-staining of a whole-mount specimen (from a naive mouse) for DCs (CD11c, green) and the basal lamina (Collagen IV, red) in the cornea was examined with a confocal microscope. A dendriform DC on the top of the BM **(A)**. A round-shaped DC that spans 13 optical sections including vertical crossing of the basal lamina, sections 8–12 **(B)**. Optical sections of A and B from 2 channel confocal imaging were digitally rendered in 3D. Arrows, BM-penetrating, round-shaped DCs **(C)**. The results are representative of two independent experiments, 3 corneas each.

To further determine if these round-shaped DCs penetrate the basement membrane (BM), we co-stained the corneas with collagen-IV and CD11c (Fig. 3). CD11c-staining showing multiple dendrites (Dendriform) spanned 7 optical sections in depth on the z-stack (each 0.9 μm) (Fig. 3A). Collagen-IV staining appeared at section level 8 where no CD11c detected, indicating that dendriform DC was not located in the same plane as the BM. (Fig. 3A, section 8). A round-shaped cell was detected in the same plane as the dendriform DC (Fig. 3B), but extended crossing BM, and ended in the stroma (Fig. 3B), spanning 13 sections in total (~1 1.7 μm). 3-D reconstruction showed that while the dendriform cells can only be seen on the top of the BM, the round-shaped cell was found at both sides of the BM (Fig. 3C, arrows).

**Figure 4:**
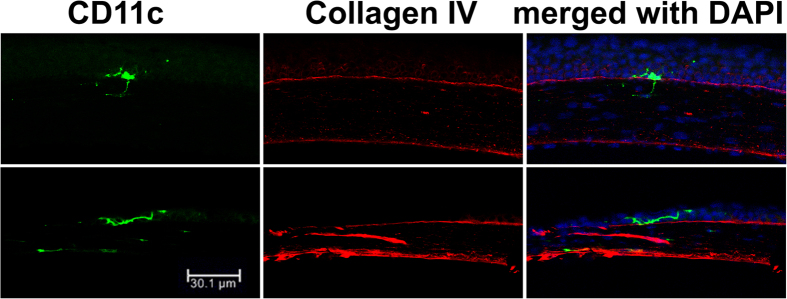
Localization of DCs within the basement membrane in mouse corneas. The cryostat sections of the normal corneas were collected and subjected to IHC analysis for DCs (CD11c, green) and the basal lamina (Collagen IV, red) with DAPI for nuclear staining and examined with a confocal microscope. The results are representatives of 3 B6 mouse corneas examined.

To further confirm the localization of DCs within the BM, we co-stained for DCs and BM on a cryostat section (10 μm) (Fig. 4). While (A) a dendriform DCa was located above the BM (B), a round-shaped DC penetrated the BM (red).

### The round-shaped dendritic cells express CD86

**Figure 5:**
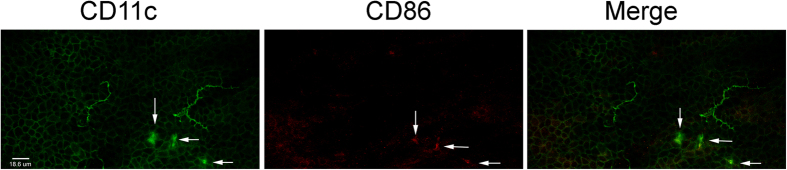
Co-localization of CD11c and CD86 in mouse corneas. Double-staining of a whole-mount mouse cornea for DCs (CD11c, green) and CD86, an activation and or maturation (CD86) was examined and photographed with a confocal microscope. Note: only round shaped CD11c positive cells were CD86 positive (arrows).

Having shown the different patterns of DC-sensory nerve association, we next investigated whether these morphologically different DCs express any activation/maturation marker(s). While dendriform DCs were CD86 negative, round-shaped DCs were CD86 positive (Fig. 5).

### Trigeminal denervation causes the loss of dendriform DCs

**Figure 6:**
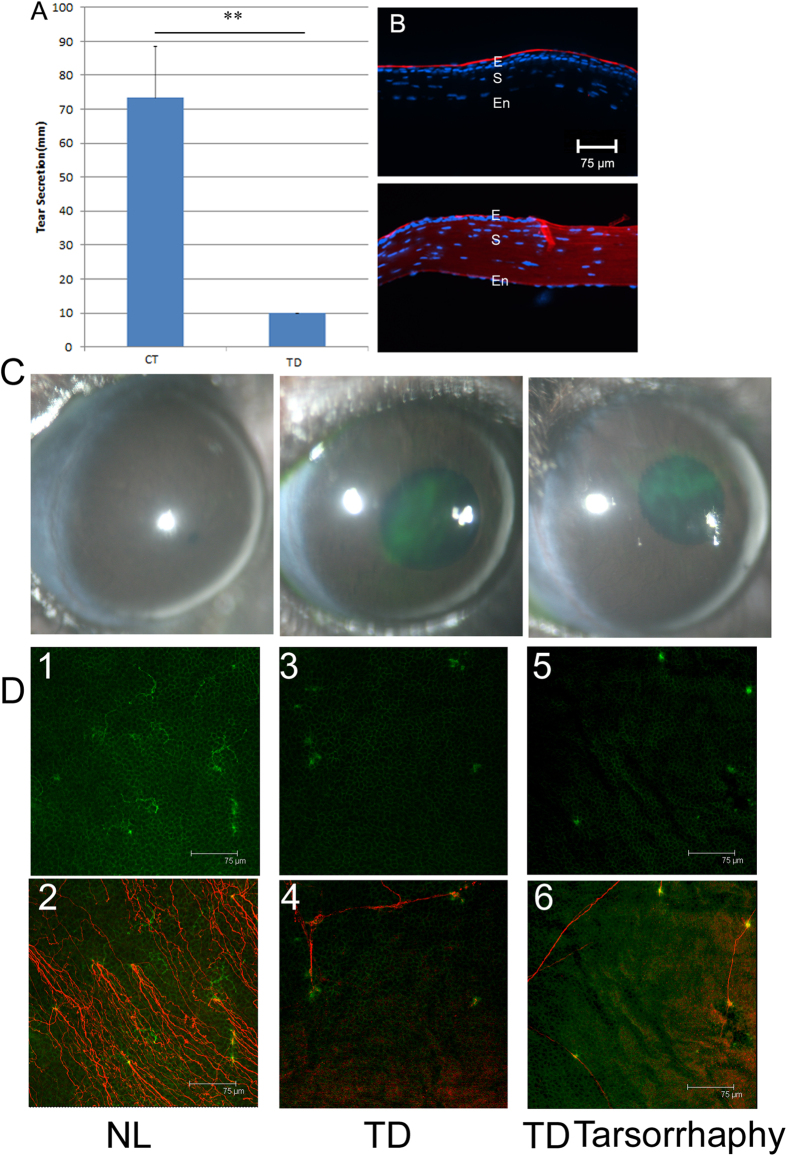
Effects of denervation on corneal DC morphology and DC-nerve interactions. The corneas were denervated using trigeminal stereotactic electrolysis (35) at dya 0. At day 1, tear secretion was measured with cotton threads (**A**). The epithelial barrier function was assessed with surface biotinylation using a crossing-link reagent sulfo-NHS-LC-biotin (**B**). E, epithelium; S, stroma; En, endothelium. The epithelial defect was examined by ocular fluorescence surface staining (**C**). Whole mount staining of β-tubulin III and CD11c near the limbal region showing the co-localization of CD11c (D1) and epithelium-innervating nerves (D2) in a control cornea or with (D3 and 4) or without tarsorrhaphy (D5 and 6) in denervated corneas (**D**). Note: the disappearance of dendriform DCs and the association of round-shaped DCs with sensory nerve fragments in denervated corneas. The results are representatives of two independent experiments, 3 corneas each.

To assess the role of sensory nerve in corneal hemostasis, trigeminal denervation was performed^24^, resulting in a marked decrease in tear secretion (Fig. 6A) and the loss of corneal sensitivity (data not shown) at 1 day post-surgery. Surface biotinylation using a crossing-linking reagent sulfo-NHS-LC-biotin^25^ showed while biotin labeling can only be found at the apical surface in the control cornea, the entire trigeminus-denervated cornea was labeled, suggesting the loss of the tight junction barrier (Fig. 6B). Figure 6B also showed edema and increased infiltration in a trigeminus-denervated cornea. Fluorescence staining of the cornea revealed epithelial defects (Fig. 6C) in the trigeminus-denervated corneas with or without tarsorrhaphy (TD sutured). While the normal cornea contained two morphologically distinct CD11c-positive cells (Fig. 6D-1,2); all CD11c-positive cells in the trigeminus-denervated cornea were round-shaped and associated with the remaining neuron fragments with (Fig. 6D-3,4) or without tarsorrhaphy (Fig. 6D-5,6).

### Depletion of DCs resulted in reduced nerve ending density and delayed post-wounding re-innervation

**Figure 7:**
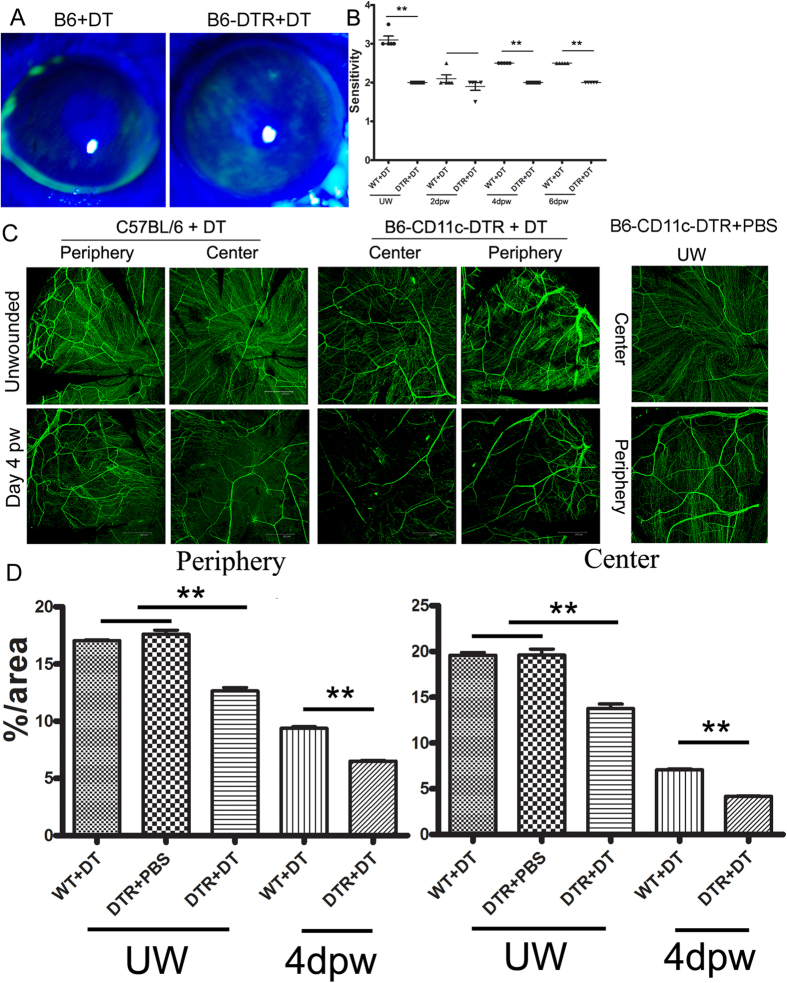
Effects of dendritic cell depletion on sensory nerve fibers/endings in normal, nondiabetic mouse corneas. DCs were depleted from B6-CD11c-DTR, but not the control B6 mice with subconjunctival injection of DT. DC-depleted and control corneas were wounded by epithelium-debridement 1 day after the first injection. Injections were repeated every 2 days after the initial injection. At day 3, unwounded corneas were stained with 0.5% fluorescein (**A**). Corneal sensitivity was measured before and 2, 4 and 6 days post-wounding (**B**). Corneas were either directly processed or wounded at 1 day post DT injection and then processed for whole mount staining of β-tubulin III at day 5 after the initial injection of DT (**C**). Note the decreased density of primarily nerve endings in both peripheral and central corneas of B6-CD11c-DTR, but not the control B6 mice at day 5 of initial DT injection. The innervation was quantitated using Image J and the results were shown as percentage of the areas covered with β-tubulin III staining at day 5 of unwounded corneas (UW) or of wounded (4 days post wounding (4 dpw)) (**D**). The results were presented as mean + SD and are representative of two independent experiments (*n* = 5 each), **p < 0.01 (Unpaired Student’s *t* test).

We next investigated the role of DCs in maintaining nerve structure and regeneration during wound healing, using diphtheria toxin (DT)-mediated DC depletion in B6-CD11c-DTR mice^26^. DC depletion resulted in fluorescence staining of the ocular surface, indicating epithelium defects (Fig. 7A). DC depletion also significantly affected corneal sensitivity in unwounded corneas and delayed its recovery during wound healing up to 6 days post-wounding (Fig. 7B). WMFM of β-tubulin III revealed marked decreases in the density of nerve endings in the unwounded corneas in B6-CD11c-DTR mice compared to WT B6 mice 5 days after the first DT injection and with B6-CD11c-DTR without injecting DT (UW, Fig. 7C,D). In healing corneas, nerve regeneration was significantly delayed at the peripheral region, with little nerve endings formed in the center of the cornea. The nerve fiber number and density was decreased and no neuronal network was observed at 4 days post-wounding in B6-CD11c-DTR mice (day 5 post-DT injection, Fig. 7C,D). Using the areas covered with β-tubulin III staining as a parameter for corneal innervation, depletion of DC significantly decreased nerve density in unwounded corneas at both central and peripheral regions and inhibited nerve regeneration at 4 days post-wounding (Fig. 7D).

## Discussion

In the present study, we report that DCs and sensory nerves co-localize within the sub-basal nerve plexus of the cornea and that their interactions are critical in corneal homeostasis after wounding. We showed the presence of two types of DCs with different morphologies and phenotypes in adult corneas: those with dendrites (CD86 negative) that interconnected with several nerve endings in the subbasal plexus, and round-shaped DCs (CD86 positive) that were co-localized with nerve fiber branching points in the subbasal plexus and that perpendicularly penetrate the BM. Both denervation and DC depletion resulted in epithelial defects with or without total tarsorrhaphy, revealing that both nerves and DCs, in the platform of the epithelium are structurally and functionally interdependent. Hence, we propose that epithelial cells, dendritic cells, and sensory nerve, act as a function unit in the cornea DC-nerve interaction plays a role in corneal homeostasis and nerve regeneration.

In the cornea, DCs have been shown to play a role in inflammation^27^,^28^, allograft rejection^29^, dry eyes^30^, and wound healing^26^. In the present study, we showed two morphologically distinct intraepithelial DCs in the unwounded cornea. Dendriform DCs in the cornea were found to be intimately in contact with sensory nerve fibers/endings, similar to those observed in the lung^22^,^31^,^32^, kidney^33^, and cornea^34^. This is expected, given the high density of subbasal nerves, as any cell in the subbasal plexus layer would be in close proximity with the nerves. A recent study using WMCM revealed that at the steady state, 75% of dermal DCs were either in direct contact with or in close proximity to sensory nerves and that these dermal DCs received signals from physical interactions with sensory nerves^34^. In human corneas with Thygeson’s superficial punctate keratitis, dendritic cells were detected in association with the subepithelial nerve plexus immediately above Bowman’s membrane and formed aggregates of mature DCs in association with subepithelial nerve fibers in the central cornea of the affected eyes as seen on confocal microscope *in vivo* (HRTII-RCM imaging)^35^. In a mouse model of herpes simplex virus keratitis, corneal DCs were shown to mediate the transmission of the virus to corneal nerves, suggesting a physical contact of DCs with epithelium and sensory nerve in infected corneas^36^. How sensory nerves might influence intraepithelial DCs in homeostatic and wounded corneas remain to be investigated.

One of the most intriguing discoveries is the co-localization of DCs with nerve fibers that vertically penetrate the basement membrane as they reach the epithelium. To date, how the sensory nerve penetrates the BM to innervate the epithelium is unclear. A recent study revealed small fenestrations on the epithelial BM of the human and rabbit corneas, which were suspected as the paths for sensory nerve fibers to innervate the epithelium^37^. In the skin, the axons that extend vertically into the epidermis clustered around the dendrites of the Langerhans cells (LCs), which are epidermal DCs^38^. The round-shaped cells observed in the resting corneas expressed CD86, suggesting they are phenotypically mature and/or activated. Our study revealed the presence of DCs along each vertical fiber that crosses the BM and branches into epithelial nerve endings, suggesting a potential role of residential DCs for epithelium innervation by not only releasing neurotrophic factors, such as CNTF^39^, but also by maintaining direct contact with the nerve as it passes through the BM. This should have implications in our understanding of the immune-nervous interplay in maintaining tissue homeostasis and in corneal response to injury and infection^40^.

The close association of sensory nerves and DCs suggest that the immune and nervous systems could influence each other functionally in the homeostatic cornea. To that end, we performed trigeminal denervation experiment and observed, in addition to disrupted tear secretion and epithelium defects which occur with or without total tarsorrhaphy, no dendriform DCs can be found in the cornea. In humans, temporary tarsorrhaphy has been used to treat persistent corneal epithelial defects including these caused by neurotrophic keratopathy^41^,^42^. Total denervation in animal model resulted in tear abnormality and epithelium defects even after tarsorrhaphy as we showed here and by others^43^. The disparity between medical treatment and animal model of denervation is likely due to the fact that in patients there are neurotrophic defects, but not complete denervation as seen in animal models. There were a few round-shaped CD11c positive cells associated with fragments remaining from the sensory nerves. It should be noted that denervation increase tissue inflammation which can result in morphological changes of DCs to be round-shaped, as showing in the cornea treated with lipopolysaccharides, resulting in dramatic cell shape change from dendriform to round-shaped near the LPS injection site^19^. Future studies to assess sensory nerve effects, likely through the release of neuropeptide substance P and/or calcitonin gene-related peptide, on DC maturation and activation in these two types of DCs are warranted.

How DCs might influence peripheral neurons remains to be elucidated. A host of neurotrophic factors termed neurotrophins, such as NGF, NT3, and CNTF, are known to be expressed in peripheral tissues for the maintenance of innervation. In the cornea, the transcription of NGF, NT-3, and BDNF was detected in *ex vivo* human corneas^44^ while others have shown that NGF was primarily expressed in the limbal epithelial cells^45^. In the accompanied study^39^, we showed that the intraepithelial DCs express CNTF, a factor known to promote sensory neuron survival and exon regeneration^46^,^47^,^48^, and that in wounded corneas, all CD11c and HMC-2 positive cells are round-shaped. More interestingly, we showed that DC depletion not only delayed epithelial wound closure^26^ but also adversely affected sensory nerve regeneration in wounded corneas. While the epithelium-wound closure was delayed for several hours, we observed significantly less nerve regeneration in DC-depleted corneas, compared to control corneas. Recently, Sarkar *et al.* reported that CD11b+GR1+ myeloid cells secrete NGF and promote trigeminal ganglion neurite growth^49^. It is possible that elevated NGF and CNTF and/or the infiltrated cells expressing them are required for proper nerve regeneration in the cornea. Further study to identify molecules, including neurotrophic factors and axon guidance ligands and receptors, responsible in mediating sensory nerve regeneration is warranted.

In summary, our data showed that intraepithelial DCs and sensory nerve axons have intimate connections and are functionally interdependent in the cornea and during epithelial wound healing.

## Materials and Methods

### Mice

Wild type C57BL6 (B6) mice (8 weeks of age; 20 to 24 g weight) and B6-DTR (B6.FVB-Tg(Itgax-DTR/EGFP)57Lan/J) mouse breeding pairs were purchased from the Jackson Laboratory (Bar Harbor, ME). B6-DTR mice carry a transgene encoding a simian diphtheria toxin (DT) receptor (DTR)-enhanced green fluorescent protein (EGFP) fusion protein under the control of the murine CD11c promoter, which makes them sensitive to DT. B6-DTR mice were bred in-house and their pups were subjected to genotyping before use. Animals were treated in compliance with the ARVO Statement on the Use of Animals in Ophthalmic and Vision Research. The Institutional Animal Care and Use Committee of Wayne State University approved all animal procedures.

### Immunostaining of Whole-Mount Corneal Tissue

Mice were euthanatized and the entire eyeball were enucleated and fixed in 4% paraformaldehyde for 10 mins. A hole was made at the retinal side, vitreous were removed and the eyeballs were fixed again at 4 °C until further processing. Cornea plus the limbus was excised under the operating microscope. Before staining, 6 radial incisions were made to allow the tissue to lay flat. Corneas were washed in PBS, incubated in 20 mM pre-warmed EDTA for 30 minutes at 37 °C to weaken epithelium-stroma adhesion and cell-cell junction to allow antibodies to penetrate into the subbasal space, and incubated with 0.025% hyaluronidase 37 °C for 1 day. For blocking, corneas were incubated in PBS containing 0.2% Triton-X, 1% bovine serum albumin, 10% serum of the host species used to generate the first antibody, or Fc blocking antibody (BD Biosciences) for 20 minutes at room temperature^50^. After blocking, the corneas were incubated overnight at 4 °C with 100 μl of mouse anti-CD11c (BD Biosciences), anti-β-tublin III (Covance) antibodies. The corneas were then washed five times in PBS and incubated with 100 μl Cy3 or FITC-conjugated secondary antibodies diluted in PBS with 1% BSA for 1 h at room temperature. This was followed by five washes in PBS. Stained corneal whole mounts were placed in Vectashield mounting medium (Vector Lab, Burlingame, CA) onto glass slides and coverslipped. Negative controls included watched IgG or mouse IgG subtypes to replace the first antibody; if the background was high, different washing, blocking, or first antibody will be tested until the background staining was undetectable. Corneal whole-mounts were examined using a confocal microscopy (TCSSP2; Leica) or Nikon ECLIPSE 90i microscopy. To quantify innervation of the corneas, Image J was used. CD11c positive nerves were highlighted using the thresholding tool of Image J. The images were converted to black-and-white images and nerve density by analyzing particles, and then calculated as the percentage of the area occupied by nerves over the total area of the analyzed image.

### Immunohistochemistry of sections

Mouse eyes were enucleated and embedded in Tissue-Tek OCT compound, and frozen in liquid nitrogen. 10 micrometer-thick sections were cut and mounted to polylysine-coated glass slides. After a 10-min fixation in 4% paraformaldehyde, slides were blocked with 10 mM sodium phosphate buffer containing 2% BSA for 1 hour at room temperature. Sections were then incubated with mouse anti-CD11c (BD Biosciences) and anti-collagen IV (EMD Millipore, MA). This was followed by a secondary antibody, FITC -conjugated goat anti-hamster and cy3- conjugated anti-rabbit IgG (Jackson ImmunoResearch Laboratories 1:100), and slides were mounted with Vectorshield mounting medium containing 4,6-diamidino-2-phenylindole dihydrochloride (DAPI) mounting media and examined under confocal microscopy. Controls were similarly treated, but the primary antibody was replaced with IgG.

### Trigeminal denervation

Animals were anesthetized with a ketamine (100 mg/mL)/xylazine (20 mg/mL) mixture. The head of each animal was shaved and disinfected with povidone iodide and ethanol. The animal was then mounted in the stereotactic frame, after which a median incision was made on the skull. The bregma (the point of conjunction of coronal and sagittal suture) was identified and chosen as a point of reference. For preliminary anatomic reference, we used a stereotaxic atlas. The skull was opened with a 25 G needle, 2 ul ethanol was injected with a microsyringe as lower (depth, 0.63 cm) and at three different locations (relative to bregma, 0.15 cm anterior and 0.08 cm lateral; 0.09 anterior and 0.10 lateral; 0.09 anterior and 0.12 lateral) on the ophthalmic trigeminal nerve. The syringe was removed, and the skin was sutured. Finally, antibiotic ointment (gentamicin sulfate) was applied to the sutural area and the treated eye. The animals were then placed on a heated pad to help the recovery. The corneal sensitivity was measured with an aesthesiometer preoperatively and postoperatively.

### Tear Secretion and Corneal Sensitivity measurements

Tear secretion was determined with phenol red–impregnated cotton threads (Zone-Quick, Tokyo, Japan). The threads were placed in the medial canthus for 1 minute and the length of the wetted part, turning red because of soaking tears, was measured. Corneal sensation was measured with an aesthesiometer (Cochet-Bonnet) in unanesthetized mice. The testing began with the nylon filament fully extended to its maximal length of 10 mm, and shortened by 1 mm each time a negative response was observed until a positive response was obtained. A positive blinking response was recorded by two observers and each test was repeated three times.

### Surface biotinylation-tight junction permeability assay

The surface biotinylation technique was performed as described by Xu *et al.*^25^ for organ cultured bovine corneas with modifications. Mouse corneas were wetted with freshly made 1 mg/ml sulfo-NHS-LC-biotin in Hank’s balanced salt solution containing 1 mM CaCl2 and 2 mM MgCl2. After 30 min incubation, the surface-labeled corneas were rinsed with PBS, embedded in OCT, frozen in liquid nitrogen, and sectioned on a cryostat at a thickness of 6 μm. The sections were fixed with ice-cold acetone and labeled with rhodamine-avidin D in PBS containing 1% BSA for 1 h. The slides were mounted and examined under a fluorescence microscope equipped with Nikon ECLIPSE 90i microscopy.

### Depletion of DCs

B6-DTR mice, with wild-type B6 mice as the control, were depleted of their DCs using a subconjunctival injection of 5 ng DT in 5 μl of phosphate-buffered saline (PBS) 24 h before; 24 and 72 h after wounding. Previous studies showed complete depletion of DC locally in the cornea^26^.

### Corneal Epithelial Debridement Wound

Anesthetized mouse corneas were first demarcated with a 2 mm trephine in the central cornea and CECs were removed with a blunt scalpel blade under a dissecting microscope^51^,^52^,^53^. Bacitracin ophthalmic ointment was applied to the cornea after surgery to prevent infection. The remaining wound was assessed using ocular surface fluorescence staining.

### Statistical analysis

Data was presented as the means ± SD. Statistical differences among three or more groups were identified using one-way ANOVA. The unpaired t-test was performed to assess for significant differences (p < 0.05) between groups. Experiments were repeated at least twice to ensure reproducibility.

## Additional Information

**How to cite this article**: Gao, N. *et al.* Intraepithelial dendritic cells and sensory nerves are structurally associated and functional interdependent in the cornea. *Sci. Rep.* **6**, 36414; doi: 10.1038/srep36414 (2016).

**Publisher’s note**: Springer Nature remains neutral with regard to jurisdictional claims in published maps and institutional affiliations.
